# The key role and research progress of endothelial cells in renal microcirculation

**DOI:** 10.3389/fmed.2026.1717495

**Published:** 2026-02-04

**Authors:** Weikang Tang, Huixia Liu, Xuan Li, Siyao Deng

**Affiliations:** School of Medicine, Tarim University, Xinjiang, China

**Keywords:** drug therapy, endothelial cells, microcirculation disorders, microvessels, renal diseases

## Abstract

**Background:**

Microcirculation disorder is the main reason for the occurrence and development of kidney diseases. Studies have shown that endothelial cells play an important role in renal microcirculation disorders. To clarify the specific function of endothelial cells on renal microcirculation and its effect after damage, and to evaluate the existing research.

**Methods:**

The reliable articles included in CNKI, PubMed, Central, Scopus, and Web of Science databases were selected as the research objects. Peer-reviewed articles and reports on endothelial cells and renal microcirculation disorders were retrieved with a literature period of nearly 15 years.

**Results and conclusions:**

We found that endothelial cells regulate the relaxation of renal microvessels, maintain the balance of microvascular perfusion and function stability, and analyze the effect of endothelial cell dysfunction on renal microcirculation, as well as the changes of endothelial cells in a variety of renal diseases, aiming to provide theoretical basis and new research direction for the prevention and treatment of renal related diseases.

## 1 Introduction

As an important system for excretion and metabolism regulation, the kidney’s microcirculation system is an indispensable structural basis for maintaining normal renal function (1). As the core element of the renal microcirculation system and the key component of the blood vessel wall, endothelial cells have an irreplaceable core role in maintaining the normal physiological state of the renal microcirculation (2). The microcirculation system is the fundamental guarantee for the realization of all functions of the kidneys. Endothelial cells actively participate in the dynamic balance regulation of renal microcirculation through a variety of complex physiological processes and molecular mechanisms, and are crucial for maintaining the stability of the internal renal environment and ensuring the normal functions of glomerular filtration, renal tubular reabsorption and secretion (3). In-depth exploration of the internal relationship and interaction mechanism between endothelial cells and renal microcirculation not only helps to reveal the microscopic mysteries of renal physiological function, but also provides a new perspective and key theoretical support for the pathogenesis analysis of various renal diseases, the innovation of early diagnosis methods and the development of precision treatment strategies, which is of profound significance to promote the development of renal medicine.

## 2 Specific functions of endothelial cells in renal microcirculation

**Figure 1:**
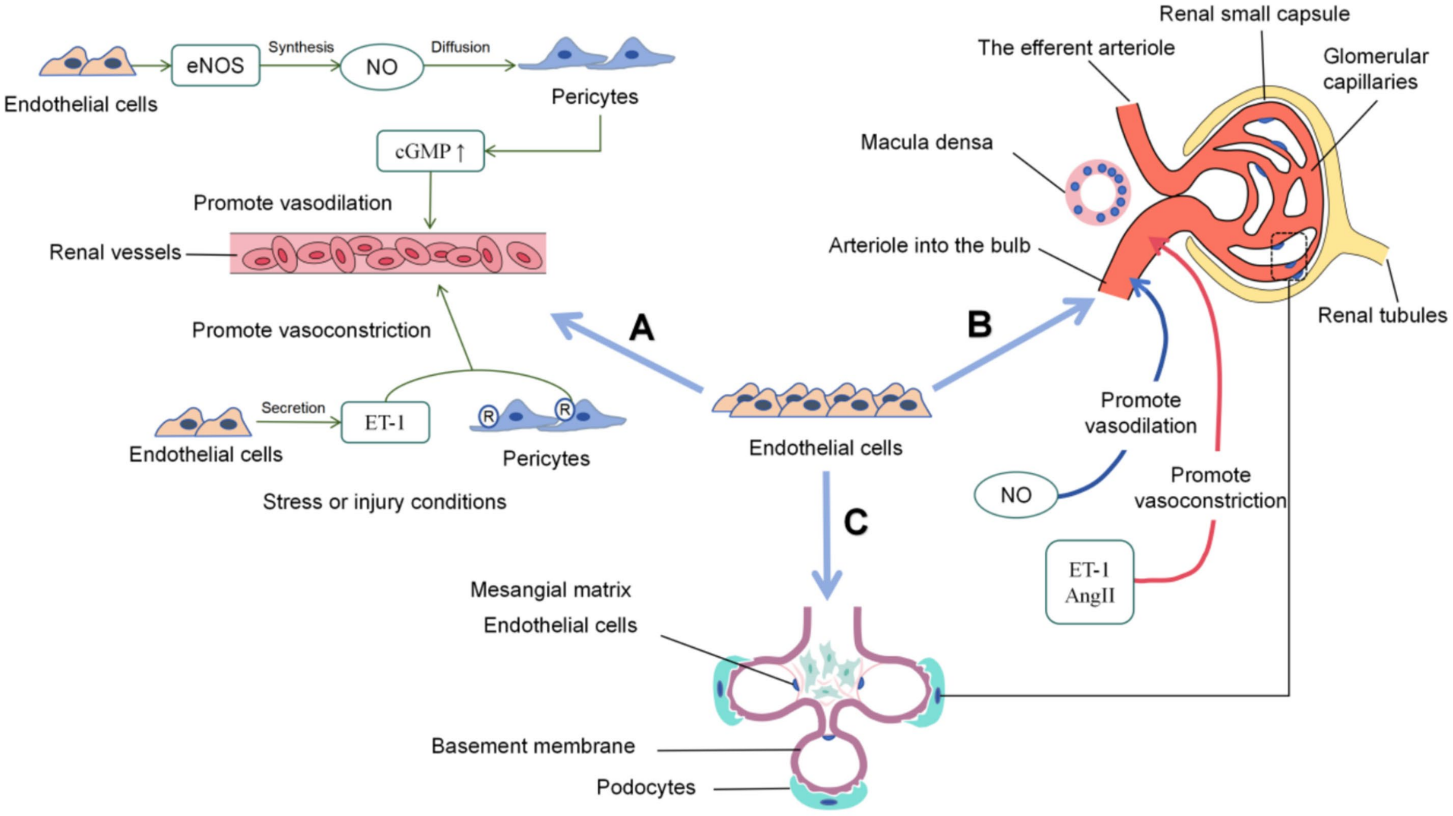
Specific functions of endothelial cells in the renal microcirculation. **(A)** Regulation of microvascular diameter and relaxation (endothelial cells synthesize eNOS to produce NO, which promotes pericyte relaxation and vasodilation; endothelial cells secrete ET-1 to induce pericyte contraction and vasoconstriction); **(B)** Regulation of renal blood flow perfusion (endothelial cells maintain perfusion stability by regulating capillary permeability and secreting vasoactive substances); **(C)** Maintenance of renal structural integrity (endothelial cells form the glomerular filtration barrier with the glomerular basement membrane and podocytes, and the surface glycoproteins form a charge-selective barrier).

Endothelial cells are the core hub for the maintenance of renal microcirculation function. As one of the organs with the highest vascular density and the richest endothelial cell subtypes in the human body, the kidney receives about 20% of the cardiac output perfusion in the resting state, and this abundant blood flow is essential for the kidney to perform its excretional and regulatory functions effectively (4). Microvascular endothelial cells form an extensive and highly organized network that ensures efficient delivery of oxygen and nutrients to renal parenchyma and clearance of metabolic waste. Any disruption of the integrity or function of these endothelial cells may result in impaired blood perfusion, triggering a series of pathophysiological reactions such as ischemia–reperfusion injury, inflammation, and fibrosis, and ultimately damaging renal function. Microvascular endothelial cells are the basic functional units of microcirculation. They cooperate with pericytes and vascular smooth muscle cells to regulate microvascular diameter and relaxation, thereby adjusting organ blood perfusion levels and functional status (5). The specific mechanism is shown in Figure 1.

### 2.1 Synergistically regulate the dilation and constriction of microvessels

In the renal microcirculation, endothelial cells and pericytes cooperate closely. Endothelial cells are sensitive to hemodynamic changes and metabolic demand signals, and accurately regulate microvascular diameter through intercellular signal transduction pathways in cooperation with pericytes (6, 7). Nitric oxide (NO), a potent vasodilator, is synthesized by endothelial nitric oxide synthase (eNOS) in endothelial cells in response to increased shear stress (8). NO diffuses into pericytes and activates soluble guanylate cyclase, leading to an increase in cyclic guanosine monophosphate (cGMP) levels, which in turn causes pericyte relaxation and promotes vasodilation (9). In contrast, endothelin-1 (ET-1) is a vasoconstrictor peptide secreted by endothelial cells under stress or injury. ET-1 binds to pericyte receptors, induces pericyte contraction and causes vasoconstriction. This dynamic interaction between NO and ET-1, together with other factors such as prostaglandins and angiotensin II, enables precise control of microvascular diameter and blood flow distribution (10, 11). When the renal blood flow increases due to body exercise, endothelial cells receive pressure and flow change stimuli, rapidly release vasoactive substances, interact with pericytes to relax microvessels and meet high flow demand. In the resting or low metabolic state, the microvascular diameter is moderately constricted to ensure stable perfusion, effectively maintain the balance of renal blood perfusion and functional stability (12).

### 2.2 The key role in renal blood perfusion

Endothelial cells perform a variety of important functions during renal filtration. Their unique structure enables it to form a filtration barrier, prevent thrombosis, regulate immune response, secrete various cytokines and transport substances essential for maintaining their own viability and nephron function—including nutritional substrates such as glucose and amino acids, regulatory ions such as calcium and potassium, and bioactive molecules such as precursors for prostaglandin synthesis—all of which collectively ensure the normal progress of renal filtration (13). Endothelial cells help maintain the stability of the glomerular filtration rate, acting by regulating the permeability of glomerular capillaries (14). Endothelial permeability is affected by various factors such as angiotensin II (AngII) and endothelin. Endothelial cells also participate in the formation of the filtration membrane—a barrier composed of endothelial cells and other cells in the glomerular capillary wall that prevents red blood cells (RBCs) and other blood cells from entering the glomerular filtrate (15). In addition, the miR-17 ~ 92 cluster derived from endothelial cells (a microRNA cluster derived from endothelial cells that participates in angiogenesis regulation) promotes angiogenesis and protects the kidney. Specific knockout of miR-17 ~ 92a in endothelial cells aggravates acute kidney injury (AKI) and reduces renal microvascular function after ischemia–reperfusion injury (IRI), while the use of miR-18a and miR-19b mimics alleviates renal ischemia–reperfusion (I/R) injury (renal ischemia–reperfusion injury, referring to secondary injury caused by the restoration of perfusion after renal blood supply is interrupted) in mice (16).

Existing studies have achieved the treatment of AKI by targeting endothelial cells. An engineered extracellular vesicle that specifically target endothelial cells have been designed and prepared. The engineered extracellular vesicles target P-selectin, the surface marker of injured endothelial cells, and deliver the loaded therapeutic functional molecules to the ischemic injured kidney. The loaded imaging molecules indicate the degree of kidney injury, realizing the integration of early diagnosis and targeted therapy of AKI (17). Endothelial progenitor cells (EPCs) transplantation has an obvious therapeutic effect on acute renal ischemia–reperfusion injury. EPCs transplantation exert different therapeutic effects through multiple pathways such as inhibiting inflammatory responses, promoting angiogenesis and repair, and promoting renal tubular cell regeneration (18–20). The application of laser speckle blood flow detection in renal microcirculation perfusion showed that microvascular endothelial cells, together with pericytes and vascular smooth muscle cells, maintain microcirculation homeostasis, and regulating the endothelial-derived vasoactive factor-related frequency band in spontaneously hypertensive rats has a protective effect on renal microvascular endothelial cells during hypertension (21, 22).

### 2.3 Maintenance of renal structural integrity

The glomerular filtration barrier is a highly specialized structure composed of endothelial cells, glomerular basement membrane, and podocytes. Endothelial cells form tight junctions and firmly adhere to the basement membrane to maintain the structural integrity of glomerular capillaries (23). Their fenestrations are small holes in the endothelial cell layer that play a crucial role in regulating the transport of water, electrolytes, glucose, amino acids, and small-molecule metabolites. The negatively charged surface of endothelial cells, composed of proteoglycan and glycoprotein, provides an additional selective layer. It repels negatively charged plasma proteins, and prevents them from filtering into the urine space (24, 25). This complex structure of endothelial cells, together with the basement membranes and podocytes, forms an efficient filtration barrier that allows precise regulation of glomerular filtration. Studies have found that co-culture of glomerular endothelial cells and podocytes has been found to significantly improve the maintenance of glomerular filtration barrier integrity, expand the surface area of podocyte foot processes and increase the thickness of glycocalyx (26, 27).

The sialic acid-rich glycoproteins (core components of the endothelial glycocalyx) on the surface of endothelial cells are important components of the charge-selective permeability of the glomerular filtration membrane (28). Studies have shown that loss of endothelial cell receptor adhesion G protein-coupled receptor F5 (ADGRF5) leads to changes in the gene expression of glomerular endothelial cells and disrupts the structure and selectivity of the glomerular filtration barrier (29). Negatively charged sialic acid-rich glycoproteins cooperate with heparan sulfate proteoglycan in the basement membrane and podocyte cleft diaphragm to generate an electrostatic repulsive fields. Increased synthesis of glomerular basement membrane components by podocytes and endothelial cells leads to accumulation of basement membrane material in the mesangium (30, 31). Disruption of this charge barrier—whether due to genetic mutations affecting the synthesis of sialic acid-rich glycoproteins or acquired injuries such as oxidative stress, inflammation or toxin exposure—will increase the permeability of the filtration membrane and lead to proteinuria, a common manifestation of glomerular injury (32, 33).

Endothelial cells are actively involved in the synthesis and maintenance of glomerular basement membrane. They secrete type IV collagen and laminin, key components of the basement membrane matrix (34). In the case of glomerular injury, endothelial cells are mobilized to initiate a repair response. They up-regulate the expression of growth factors and matrix metalloproteinases—enzymes crucial for the remodeling and regeneration of damaged basement membranes. However, if the injury is severe or chronic, abnormally regulated repair processes lead to excessive deposition of extracellular matrix components and the development of glomerular sclerosis and tubulointerstitial fibrosis (35, 36).

Endothelial cells are the core of renal microcirculation homeostasis, and their physiological functions are mainly reflected in three aspects: synergistically regulating microvascular dilation and contraction with pericytes, maintaining stable renal blood perfusion by regulating vascular permeability and secreting vasoactive substances, and maintaining the structural integrity of the glomerular filtration barrier through intercellular interactions and matrix synthesis.

## 3 The impact of endothelial dysfunction on renal microcirculation

Endothelial dysfunction refers to a pathological state of endothelial cells caused by various endogenous and exogenous pathogenic factors, characterized by damaged tight junction integrity, reduced eNOS activity, overexpression of immune adhesion molecules, and imbalanced secretion of bioactive substances. As the core of renal microcirculation regulation, endothelial dysfunction directly triggers a series of abnormalities in renal microcirculation, which is an important initiating link in the progression of renal diseases.

### 3.1 Results in increased vascular permeability

When endothelial cells are damaged, the integrity of their tight junctions and barrier function is destroyed, leading to abnormal increases in vascular permeability (37). A large amount of protein and fluid seeps into the renal interstitium: On the one hand, this directly leads to interstitial edema in the kidneys, increasing local tissue pressure and mechanically compressing the renal tubules and blood vessels, severely hindering the normal blood perfusion of the renal microcirculation (38). On the other hand, the massive loss of plasma proteins significantly changes the blood colloid osmotic pressure, further interfering with the balance of renal filtration and reabsorption function, leading to the gradual decline of renal function, and eventually lead to the occurrence and development of kidney disease (39, 40). Vascular permeability maintains the exchange between blood vessels, tissues, and organs. Endothelial cell receptors, including vascular cell adhesion molecule (VCAM), intercellular adhesion molecule (ICAM), vascular endothelial growth factor receptor (VEGFR-2), etc., have important functions in homeostatic and pathological environments. Increased vascular permeability is related to endothelial integrity. In diabetes and its complications, white blood cells will attach to endothelial cells and migrate during inflammation and pathogenesis, causing damage to endothelial function and leading to blood circulation dysfunction (41, 42). In some autoimmune kidney diseases, autoantibodies attack endothelial cells. For example, in lupus nephritis, anti-endothelial cell antibody (AECA) binds to endothelial cell surface antigens and disrupts the expression of tight junction protein occludin, resulting in increased vascular permeability and renal interstitial edema compressing microvessels, eventually leading to microcirculation disorders (43).

### 3.2 Influence the secretion of bioactive substances

Endothelial dysfunction is often accompanied by an imbalance in the secretion of bioactive substances. Maintenance of appropriate levels of vascular endothelial growth factor (VEGF) is essential for renal microcirculation homeostasis - VEGF is an endothelium-only growth factor that promotes proliferation, differentiation, and survival of endothelial cells, mediates endothelium-dependent vasodilation, and is involved in the formation and maintenance of endothelial fenestrations in glomerular capillaries. When endothelial function is impaired, the expression of VEGF becomes unbalanced: in the pathogenesis of diabetes and its complications, the expression of VEGF and its receptor is upregulated, and its excessive secretion lead to abnormal proliferation and disorder of renal vessels, destroying the normal structural and functional integrity of renal vessels (44). VEGF deficiency impairs the repair and regeneration ability of blood vessels, making it difficult for the kidney to achieve effective self-repair after injury (45).

In addition, when endothelial eNOS activity was inhibited or its expression was downregulated, NO synthesis was markedly reduced. NO is a powerful vasodilator and anti-inflammatory signaling molecule that maintains renal vascular status and regulates glomerular microcirculation by altering the tone of afferent arteriolar and mesangial cells (46). Insufficient synthesis of NO restricts renal vasodilation, increases vascular resistance, and then reduces renal blood flow, causes glomerular ischemia and hypoxia injury, and seriously affects glomerular filtration function (47, 48).

### 3.3 Immune response imbalance

**Figure 2:**
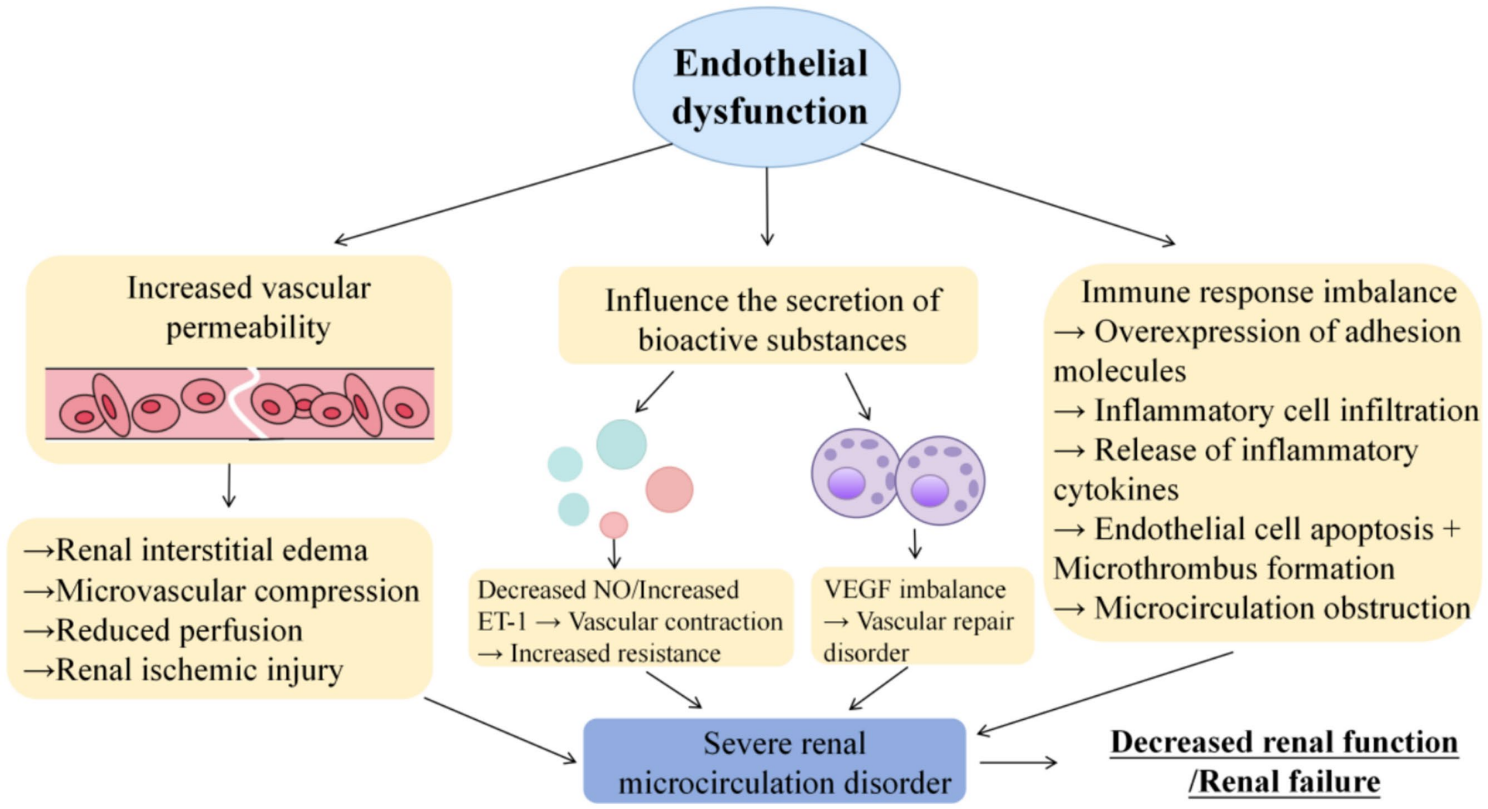
Effects of endothelial dysfunction on renal microcirculation.

Renal endothelial cells are involved in maintaining the filtration properties of the glomeruluar filtration barrier and podocyte architecture. Vascular endothelial cells support the renal vasculature, and endothelial dysfunction is an important trigger of uncontrolled local immune responses in the kidney (49). Abnormally activated endothelial cells overexpress immune adhesion molecules (such as ICAM-1, VCAM-1), and inflammasomes initiate innate immune responses, which have become important mediators of endothelial dysfunction. Vascular endothelial cells attract a large number of immune cells (such as neutrophils and macrophages) to infiltrate renal tissue through these adhesion molecules. After activation in renal tissue, these immune cells release a series of inflammatory cytokines such as IL-1, IL-6 and TNF-*α*. These inflammatory factors directly phosphorylate endothelial tight junction proteins, disrupt barrier function, and induce endothelial cell apoptosis (50, 51). In addition, it promotes platelet aggregation and microthrombosis, which further aggravates microcirculation obstruction. Glomerular endothelial cells form transcellular pores that facilitate the transport of molecules and ions through the blood vessels and mediate immune responses through leukocyte migration. In the development of microcirculation disorders, inflammatory mediators can not only directly damage endothelial cells, destroy the normal structure and function of renal tissue, but also damage renal vascular permeability, aggravate microcirculation disorders, form a vicious cycle, accelerate the pathological process of renal disease, and eventually leads to severe damage or even failure of renal function (52, 53). The specific mechanism of action is shown in Figure 2.

Endothelial dysfunction affects renal microcirculation through three core pathways: increased vascular permeability leading to interstitial edema and microvascular compression, imbalance of bioactive substances (NO/VEGF/ET-1) secretion leading to abnormal vascular tone and repair disorders, and imbalance of immune response leading to inflammatory infiltration and microthrombosis. These pathways interact with each other, leading to decreased renal microcirculation perfusion, structural damage and dysfunction, which lays the pathological foundation for the occurrence and development of kidney diseases.

## 4 Effects of endothelial cell injury on kidney disease

Endothelial cell injury-induced renal microcirculation disorder is a common pathological basis for various renal diseases. Different types of renal diseases have specific manifestations of endothelial cell injury, but they all lead to disease progression through disrupting the homeostasis of renal microcirculation.

### 4.1 Acute kidney injury

Renal vascular injury play an important role in the early pathogenesis of AKI. Renal blood flow in the kidney is mainly through the change of preglomerular vascular tone of afferent arterioles, maintaining renal perfusion pressure within the range of 60–100 mmHg. A variety of neurohormonal processes are involved in the regulation of renal microcirculation. In AKI, various pathogenic factors such as ischemia–reperfusion injury and nephrotoxic drug effects directly or indirectly cause endothelial cell damage, which then participates in the occurrence and development of AKI by affecting tension-related pathways (54). The balance of vasoactive substances secreted by damaged endothelial cells is disrupted: NO synthesis decreases and endothelin secretion increases, leading to strong renal vasoconstriction (55). The rapid decrease of renal microcirculation perfusion, persistent hypoxia, and inflammatory processes during reperfusion lead to continuous injury and dysfunction of renal tubular endothelial cells, and renal tissue can not receive sufficient blood supply, resulting in the rapid aggravation of ischemic–hypoxic injury (56, 57). Endothelial cell injury also causes coagulation imbalance and promotes the formation of microthrombi in renal blood vessels, further obstructing the vascular lumen and aggravating renal ischemia and hypoxia, which worsens the condition of sepsis-induced AKI (58). In addition, thromboinflammation in glomeruli and peritubular capillaries is an important pathogenic mechanism of AKI in sepsis-induced coagulopathy (59). Renal ischemia–reperfusion induces AKI. Scavenging free radicals and hydroperoxides, inhibiting inflammation and thrombosis effectively prevent the apoptosis of vascular endothelial cells and renal tubular epithelial cells, and improve the stability of renal cells and blood microenvironment (60). Endothelial cell injury also contributes to the progression of various glomerular diseases. IgA nephropathy (IgA glomerulonephritis) is the most common primary glomerulonephritis and the leading cause of renal failure worldwide. Immune complex deposition and mesangial-endothelial cell crosstalk contribute to elevated endothelial cell permeability, impaired integrity or dyslocalization of glomerular endothelial/filtration barrier proteins (e.g., tight junction proteins occludin and claudin, as well as the adhesion protein VE-cadherin), and recruitment of inflammatory cells—all of which collectively exacerbate renal injury (61). Acute glomerular lesions in IgA nephropathy are accompanied by glomerular capillary damage and endothelial cell loss, which lead to hematuria, proteinuria and renal insufficiency. The morphological feature of impaired glomerular capillaries in acute glomerular lesions is the separation of CD34^+^ endothelial cells from the glomerular basement membrane (62).

### 4.2 Chronic kidney disease

CKD is a long-term progressive renal disease. The persistence of metabolic disorders such as hypertension and diabetes, as well as abnormal hemodynamic status, will cause chronic and continuous stimulation and damage to endothelial cells (63). With the progression of the disease, renal oxidative stress induces vascular endothelial dysfunction, which leads to a series of pathological changes such as impaired vasodilation function, persistently high expression of immune molecules in chronic inflammatory state, and abnormal regulation of angiogenesis-related factors such as VEGF. This conclusion has been confirmed in animal experiments (64, 65). Clinical cohort studies have shown that elevated VEGF levels are positively associated with the risk of developing CKD, especially in patients with severe CKD (66). These changes together lead to gradual hardening and narrowing of renal blood vessels, gradual loss of autoregulation ability of renal microcirculation, and continuous decline of glomerular filtration rate, eventually leading to renal failure (67). Studies have found that glomerular endothelial cell-derived miR-192-5p and podocyte-derived miR-378a-3p are up-regulated in the urine and glomeruli of patients with idiopathic membranous glomerulonephritis, while nephronexin in the glomeruli is decreased, indicating that loss of nephronexin in the glomerular basement membrane of patients affects the progression of membranous glomerulonephritis (68). Endothelial cells play an important role in the early stage of the disease. Glomerular capillary damage can be seen in idiopathic membranous nephropathy, and glomerular endothelial cell damage and focal segmental glomerulosclerosis accelerate its pathogenesis (69). Galactose-deficient immunoglobulin A1 (Gd-IgA1) plays a key role in the development of IgA nephropathy. Studies on nude mice injected with Gd-IgA1-IgG immune complexes (Gd-IgA1-IgG ICs) have shown that Gd-IgA1-IgG ICs change the glycocalyx of renal microvascular endothelial cells, activate endothelial cells, and accelerate the production of adhesion factors and pro-inflammatory cytokines in glomerular endothelial cells (70). This process further promotes platelet aggregation and microthrombus formation, leading to insufficient glomerular microcirculation perfusion and accelerating CKD progression.

### 4.3 Diabetic nephropathy

DN is one of the common and serious microvascular complications of diabetes (71). Clinical studies have shown that in the prediabetic stage, insulin resistance, obesity-related inflammation and metabolic disorders lead to endothelial relaxation and fibrinolytic dysfunction, thereby increasing the risk of kidney disease (72). In the diabetic state, long-term hyperglycemia, oxidative stress and accumulation of advanced glycation end products (AGEs) cause severe damage to endothelial cells through AGE/RAGE, AMPKα, NRF2 and other pathways. AGE/RAGE pathway directly destroys the endothelial barrier, induces the degradation of tight junction proteins between endothelial cells, and increases vascular permeability. It also activated the NF-κB pathway, promoted the release of pro-inflammatory factors (TNF-*α*, IL-6) and adhesion molecules (VCAM-1), and aggravated the injury. In diabetic state, hyperglycemia and AGEs can inhibit the activation of AMPKα, reduce glucose uptake, induce endothelial cells to differentiate into mesenchymal cells, and destroy the normal structure of vascular endothelial cells. Inhibition of NRF2 pathway can cause ROS accumulation in endothelial cells, destroy the structure of endothelial cells, and aggravate the inflammatory response (73, 74). The glucose transport function of endothelial cells is impaired, leading to disorders of intracellular glucose metabolism. Hyperglycemia up-regulate chronic inflammatory markers and promote the production of reactive oxygen species (ROS). These highly oxidative substances directly damage the cell membrane, organelles, and biological macromolecules such as nucleic acids of endothelial cells, destroying the structural and functional integrity of cells, and ultimately leading to microvascular dysfunction. This conclusion has been confirmed in *in vitro* cell experiments (75). Meanwhile, AGEs engage with surface receptors on endothelial cells, with the activated PI3K/AKT and MAPK pathways acting as core signaling cascades that mediate endothelial injury, inflammatory activation, and vascular dysfunction. Persistent AGE stimulation elicits excessive activation of the PI3K/AKT pathway, which phosphorylates endothelial tight junction proteins and VE-cadherin, promoting the endocytosis, degradation, or subcellular mislocalization of these barrier proteins. This directly impairs interendothelial junctional structures and leads to enhanced vascular permeability. Additionally, AGE-induced oxidative stress and cellular damage inhibit NRF2 nuclear translocation, diminish the synthesis of antioxidant enzymes (e.g., SOD and GSH), and cause intracellular ROS accumulation—effects that further damage endothelial cells and induce their apoptosis through the activation of apoptotic proteases such as Caspase-3 (76). In addition, the antithrombotic ability of endothelial cells in DN is significantly reduced, and the blood is in a hypercoagulable state, which is prone to thrombosis, further aggravating renal microcirculation disorders and promoting the rapid progression of the disease to end-stage renal disease (77). Studies have shown that AGEs increased the expression and secretion of KIT ligand (KITLG) in renal endothelial cells. Both AGEs and KITLG promoted endothelial-mesenchymal transition of endothelial cells, and further increased the permeability of glomerular endothelial cells (GECs) through the AKT extracellular signal-regulated kinase pathway (78). The specific mechanisms of action are shown in Table 1.

**Table 1:** Effects of endothelial cell injury on kidney disease.

Kidney disease	Result	Time	References
Acute kidney disease (AKI)	Normal endothelial cells maintain a balance between NO secretion (vasodilation) and endothelin (vasoconstriction), and the imbalance directly triggers decreased renal blood flow after injury	2009	(55)
	The damage of endothelial cells destroys the self-regulation ability of microcirculation, and the amplification of inflammation during reperfusion further aggravates renal ischemia and hypoxia	2018	(56)
	Normal endothelial cells inhibit thrombosis, anticoagulant function is lost after injury, and thrombotic inflammation obstructs capillaries	2024	(59)
	Endothelial cells are the outer layer of the filtration barrier. After injury, barrier integrity is destroyed and immune complexes are abnormally deposited	2023	(61)
	The loss of endothelial cells causes the capillary wall to break down, and red blood cells and proteins leak out to form hematuria and proteinuria	2016	(62)
Chronic kidney disease (CKD)	Continuous oxidative stress in CKD directly damages endothelial function, forming a vicious cycle of “oxidative stress → endothelial injury → renal function decline”	2024	(64)
	VEGF is secreted by endothelial cells and regulates microvascular homeostasis. Endothelial injury in CKD leads to abnormal increase of VEGF and aggravates microvascular disease	2023	(66)
	Endothelial cells are the core carriers of microcirculation regulation. After injury, the regulatory ability of endothelial cells is lost, and the glomerular filtration rate is progressively decreased	2019	(67)
	Endothelial cell-derived micrornas regulate podocyte function, and abnormal expression of miRNA after injury destroys intercellular cooperation	2021	(68)
	Endothelial cell injury is one of the initial factors of focal segmental glomerulosclerosis, and they promote each other	2015	(69)
	Immune complexes directly damage glomerular endothelial cells and induce the release of inflammatory factors	2022	(70)
Diabetic nephropathy(DN)	Insulin resistance and metabolic disorders in the early stage of DN directly affect endothelial function, and abnormal relaxation and fibrinolysis are the harbingers of microangiopathy	2020	(72)
	The core pathogenic factors of DN (hyperglycemia, AGEs, oxidative stress) all target vascular endothelial cells	2021	(73)
	High oxidative active substances destroy the structural integrity of endothelial cells, resulting in increased microvascular permeability and abnormal blood flow	2014	(75)
	AGEs aggravate renal fibrosis by mediating inflammatory activation and matrix proliferation through endothelial cell receptors	2015	(76)
	AGEs in DN induce the transition of endothelial cells to mesenchymal cells, which both disrupt glomerular endothelial permeability and promote fibrosis	2022	(78)

## 5 Strategies to target endothelial cells for the treatment of kidney disease

### 5.1 Modern drug therapy

Angiotensin converting enzyme inhibitors (ACEI) and angiotensin receptor blockers (ARB) are widely used in the first-line treatment of kidney diseases. Their mechanism of action is mainly to inhibit the renin-angiotensin system (RAS), reduce the production of angiotensin II, and effectively improve endothelial cell function (79, 80). Specifically, these drugs alleviate oxidative stress injury in endothelial cells, restore the bioavailability of NO, and improve vasodilation function (81–84). At the same time, they inhibit endothelial cells from secreting pro-inflammatory factors (such as TNF-*α*, IL-6) and pro-fibrotic factors, delay the fibrosis process of renal disease, and protect renal function (85). In addition, statins, despite being well-recognized lipid-lowering drugs, not only reduce cholesterol but also exert renoprotective effects. These agents act as 3-hydroxy-3-methylglutaryl-coenzyme A (HMG-CoA) reductase inhibitors, regulating endothelial cell cholesterol metabolism, enhancing eNOS activity, stabilizing endothelial function, and improving renal microcirculation—ultimately playing an auxiliary role in the treatment of kidney diseases (86–88).

### 5.2 Cell therapy and gene therapy

As an emerging cell therapy strategy, EPCs transplantation has brought new prospects for the treatment of renal diseases (89). EPCs have unique differentiation potential—they differentiate into mature endothelial cells and homing to the site of kidney injury under the guidance of specific chemokines, actively participating in the repair and regeneration of blood vessels, thereby effectively improving renal microcirculation (90). Currently, EPC-derived extracellular vesicles protect glomerular endothelial cells from apoptosis by reducing oxidative stress and prevent leukocyte adhesion by inhibiting the expression of adhesion molecules (ICAM-1, VCAM-1, E-selectin) (91). Methods such as peripheral intravenous infusion or local injection of EPCs into kidney tissue have achieved certain therapeutic effects in animal experiments and some clinical trials, showing broad application prospects, but they still face challenges such as low cell survival rate and insufficient homing efficiency (92–94).

**Figure 3:**
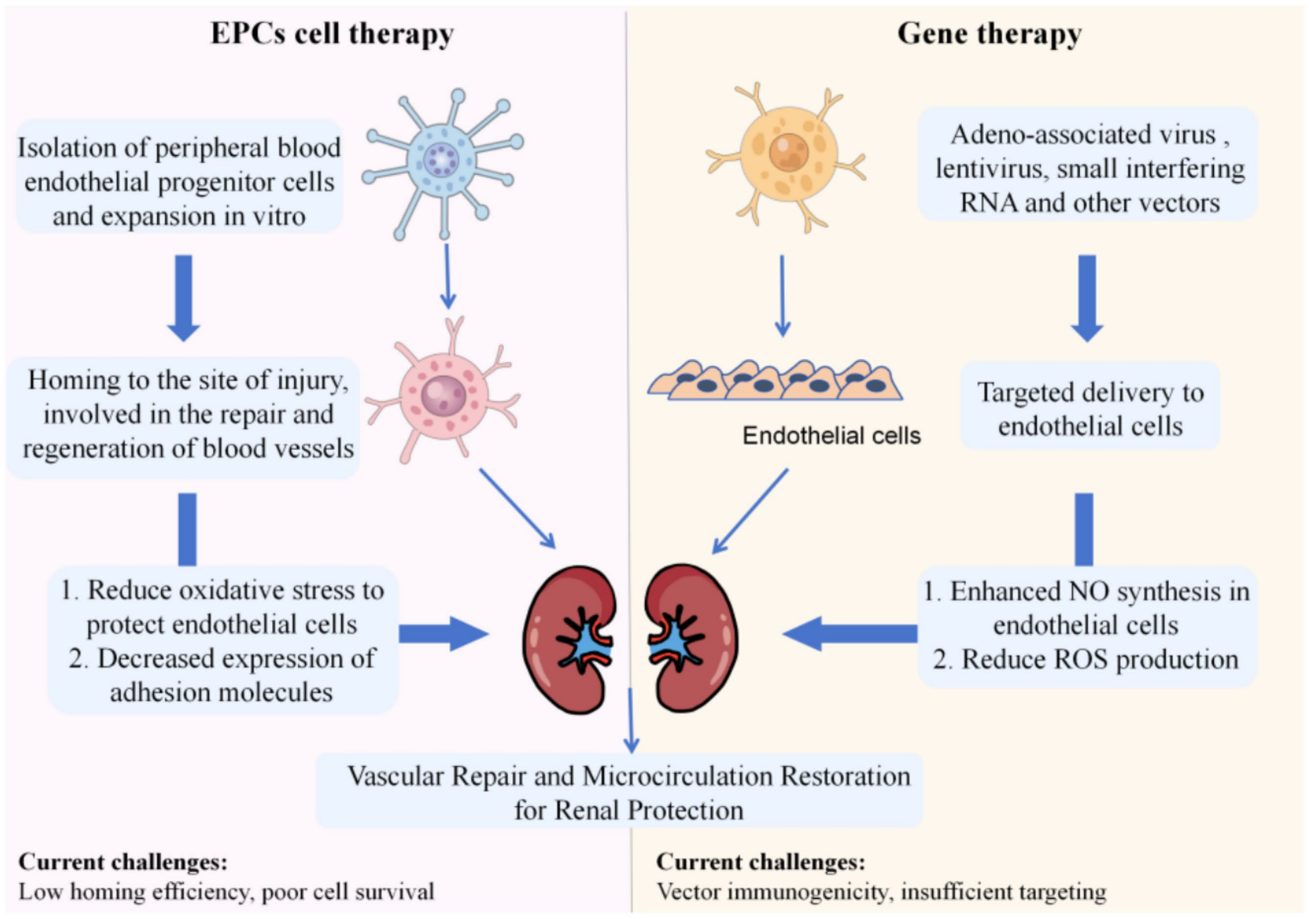
Schematic diagram of cell therapy and gene therapy targeting endothelial cells left.

Gene therapy uses modern molecular biology technology to introduce specific functional genes into endothelial cells through vectors such as adeno-associated virus (AAV), lentivirus, or small interfering RNA (siRNA). For example, introducing the eNOS gene enhance the ability of endothelial cells to synthesize NO, improve endothelial-mediated vasodilation function, and reduce the production of ROS in renal blood vessels (95–97). Introduction of anti-inflammatory and anti-apoptotic genes reduce endothelial cell injury and inflammatory response, precisely regulate endothelial cell function at the genetic level, and promote the recovery of kidney disease (98–101). Although gene therapy is still in the research and exploration stage, it has shown great potential therapeutic value, but it also has problems such as vector immunogenicity and poor targeting. As shown in Figure 3.

### 5.3 The effective active ingredient of herbal medicine treatment

Traditional Chinese herbal medicine has a long history and unique advantages in the treatment of kidney diseases. Some components of traditional Chinese medicine have been confirmed by modern medical research to have significant effects in protecting endothelial cells and improving renal microcirculation (102). For example, Astragalus polysaccharides in *Astragalus membranaceus* exert endothelial cell protection through multiple pathways such as anti-oxidative stress and immune function regulation. It scavenge excessive free radicals in cells, reduce oxidative stress damage to endothelial cells, regulate the body’s immune balance, inhibit the attack of excessive immune activation on endothelial cells, and promote the release of NO, thereby improving renal blood perfusion (103, 104). Tanshinones in *Salvia miltiorrhiza* have multiple pharmacological effects, such as anti-platelet aggregation, inhibition of vascular smooth muscle cell proliferation and anti-inflammation (105). Tanshinone IIa effectively reduce the inflammatory response of endothelial cells, improve renal vascular endothelial function, and exert a comprehensive and effective regulatory effect on renal microcirculation through the synergistic effect of multiple targets and multiple pathways, which provides a new option for the treatment of kidney diseases (106–108).

## 6 Discussion

Endothelial cells play an absolute core role in the normal operation of renal microcirculation, and their integrity and stability are crucial for maintaining renal physiological functions (109). Endothelial cell dysfunction is closely related to the occurrence and development of various renal diseases such as AKI, CKD, and DN They interact with each other and form a complex pathophysiological network (110). An in-depth understanding of the intrinsic relationship and mechanism between endothelial cells and renal microcirculation opens up a new direction for renal disease research, and also provides a wealth of potential targets and treatment strategies for clinical treatment (111). Targeting endothelial cells through drug therapy, cell therapy, gene therapy and other means is expected to break through the limitations of traditional renal disease treatment (traditional renal disease treatment mainly includes blood pressure lowering (calcium channel blockers), blood sugar lowering, diuretics etc., which are difficult to target repair endothelial damage and microcirculation disorders), provide patients with more accurate and effective treatment, and improve the prognosis and quality of life of patients (112, 113).

### 6.1 The unsolved problems and challenges in this field

Despite the significant progress made in related research, there are still many unresolved problems and challenges: First, the long-term efficacy and safety of various treatment strategies need to be evaluated—for example, the long-term adverse reactions of EPCs transplantation and gene therapy in clinical applications are still unclear, and large-sample and long-term follow-up studies are lacking; Second, the combination optimization of different treatment methods needs to be explored—how to combine modern drugs, cell therapy and traditional Chinese medicine components to achieve synergistic therapeutic effects and reduce side effects; Third, the more precise molecular regulatory mechanism of endothelial cells in renal diseases needs to be clarified—such as the specific molecular mechanism of the synergistic effect between endothelial cells and mesangial cells, how the cytokine network secreted by endothelial cells precisely regulates local hemodynamics and coagulation processes, and the cytokine network that initiates the fibrosis process after endothelial cell damage needs to be further analyzed.

### 6.2 Potential solutions and future research priorities

To address the above challenges, the following potential solutions are considered: Carry out large-sample clinical studies to evaluate the long-term efficacy and safety of targeted endothelial cell therapies. While this strategy is inherently challenging—requiring extensive patient recruitment, substantial time investment (typically years for follow-up), significant financial resources, and multi-center collaboration to ensure sample representativeness—it remains valuable for validating clinical translatability. Key considerations for implementation include leveraging existing clinical trial networks, optimizing inclusion/exclusion criteria to streamline recruitment, and integrating real-world data to complement prospective studies, thereby balancing scientific rigor with practical feasibility. Develop targeted delivery systems to improve the targeting and bioavailability of drugs/genes/cells. This field has witnessed remarkable progress: for instance, monoclonal antibody-conjugated carriers (e.g., anti-VEGFR2 antibody-modified liposomes) have been shown to selectively target renal endothelial cells, reducing off-target effects (17). Meanwhile, nanoparticle-based systems (lipid nanoparticles) enhance the efficacy of targeted delivery and co-delivery of drugs, promote intracellular uptake, and have high manufactuability and good safety (114). These strategies hold great promise for overcoming the limitations of conventional therapies, such as poor tissue penetration and systemic toxicity. Integrate multi-omics technologies to systematically analyze the molecular regulatory network of endothelial cells in renal microcirculation. This holistic approach can reveal previously unrecognized signaling pathways and interaction nodes, providing a basis for precise identification of therapeutic targets.

This review offers novel insights: First, the synergistic regulatory network among endothelial cells, mesangial cells, and pericytes is pivotal for maintaining renal microcirculation homeostasis, and the dysregulation of this network serves as the core driver of renal disease progression. Second, the crosstalk between endothelial cells and other renal cell populations (e.g., podocytes) is dynamically regulated by both direct cell–cell contact and paracrine signaling, which modulates microcirculatory function and disease susceptibility.

Future research should focus on three directions: (1) Elucidating the specific molecular mechanisms of interactions between endothelial cells and other renal cells (mesangial cells, podocytes and pericytes), including the identification of key signaling molecules and their downstream effectors; (2) To clarify the role of endothelial cell-derived exosomes and micrornas in the regulation of renal microcirculation, especially in intercellular communication during disease progression; (3) Develop novel drugs or therapeutic strategies targeting the endothelial-microcirculation axis and accelerate their preclinical validation and clinical translation through interdisciplinary collaboration, such as combining delivery system innovation with omics guided target discovery.
